# Supplementary material for: Health Effects of a 12-Week Web-Based Lifestyle Intervention for Physically Inactive and Overweight or Obese Adults: Study Protocol of Two Randomized Controlled Clinical Trials
Source: Int J Environ Res Public Health. 2022 Jan 26;19(3):1393. doi: 10.3390/ijerph19031393 (PMC8835149; doi:10.3390/ijerph19031393)
Supplement: Supplementary file 1 [file ijerph-19-01393-s001.zip › File S1.pdf]

Albert-Ludwigs-Universität Freiburg

**Research project**
**Evaluation of web-based health programs of Techniker Krankenkasse (Fitness)**
Clinical trial

STUDY INFORMATION

November 2019

Dear prospective student,

You may be planning to take part in the clinical trial of the evaluation of web-based health programs of the Techniker Krankenkasse (Fitness). With this information letter, we would like to inform you comprehensively about the aims, contents, and data protection concerns of the clinical trial, which we will also communicate to you verbally.

The clinical trial is being conducted on behalf of the Techniker Krankenkasse by the Department of Sport and Sport Science (DoSS) of the University of Freiburg in cooperation with the Section of Health Care Research and Rehabilitation Research (SEVERA) of the Medical Center of the University of Freiburg and the technical implementation partner Vilua Healthcare GmbH. If you have any questions about the trial, please contact us at any time by phone or e-mail (Judith Brame, +49 761-203-54087, tk-studie@sport.uni-freiburg.de).

Background and aims of the clinical trial

Physical inactivity is one of the most significant health risk factors. Current research shows that physical inactivity leads to an increased mortality and morbidity risk of diseases, especially diseases of affluence, such as obesity, blood sugar, and various metabolic disorders. At the same time, it has been convincingly proven that sufficient physical activity significantly reduces this health risk and has many positive effects on important health parameters, such as body measurements, blood pressure, blood values, and vascular function.

Consequently, physical activity is to be considered an essential component of individual and sustainable health programs. This has been comprehensively confirmed in numerous studies conducted in personal contact (face-to-face) health programs. In the course of modern digitalization, it is, therefore, necessary to investigate to what extent this health-promoting potential of physical activity can be transferred to health programs that are delivered via the internet (web-based). Previous studies indicate that these programs are also successful, but at the same time, there is still a considerable need for research.

The Techniker Krankenkasse offers various web-based health programs to increase physical activity and fitness (in short: “Fitness”) and would like to evaluate them scientifically within the present research project. The focus is on a modern, interactive web-based program and a classic, non-interactive web-based program. Both web-based programs are recommendations for action and contents of scientifically based guidelines that support you in achieving your physical activity and fitness goals. The clinical trial aims to examine these two health programs - in addition to the online survey - from a sports and nutritional medicine perspective. This is to check the effectiveness of these health programs on health behavior (physical activity, dietary behavior) and important health parameters (physical fitness, body measurements, blood pressure, blood values, vascular function).

Participation in the clinical trial

In principle, healthy women and men aged between 18 and 65 years with a body mass index (BMI) between 18.5 and 34.9 kg/m^2 can participate in the clinical trial. If you have any questions about the BMI or how it is calculated (=kg/m^2), please contact the study staff. If you have any existing illnesses or health impairments (at rest/under stress), you must submit a medical certificate of fitness for sport. Furthermore, you should not exercise more than 60 min/week, and you should not be pregnant. As the allocation of the study groups is randomized, you must be willing to be allocated to the intervention or control group. In addition, participation in the trial is possible even if you are not insured with Techniker Krankenkasse.

For successful participation in the clinical trial and completion of all scheduled medical examinations, you will receive the activity tracker Fitbit Charge 3™ worth € 150 as an expense allowance. If you terminate the trial prematurely, you must return it.

Procedure and contents of the clinical trial

If you would like to register for the clinical trial, please confirm your declaration of consent on the following pages and provide your contact details and time availability. We will then try to reach you by phone or e-mail as soon as possible. If we cannot reach you, we will ask you to get back to us as soon as possible. You can reach us by telephone from Monday to Friday from 8 a.m. to 6 p.m. at +49 761-203-54087 or by e-mail at any time at tk-studie@sport.uni-freiburg.de. At the bottom of this and the following page, you have the option of saving and printing the study information and the consent form. In addition, these documents are automatically saved in your health program and can be accessed there at any time.

When you are contacted, your ability to participate will first be checked using a short questionnaire following the inclusion criteria mentioned above, and an appointment will be made with you for the first medical examination. If you have successfully participated in this examination, you will then complete either the modern, interactive web-based program (intervention group) or the classic, non-interactive web-based program (control group) to increase physical activity and fitness over a recommended period of at least 12 weeks. The respective program will be activated online seven days after the first medical examination and can be used by you during the entire examination period (probably until autumn 2021).

After 12 weeks and further 6 and 12 months, you will complete a second, third and fourth examination in addition to the online survey. The study procedure can be seen below:

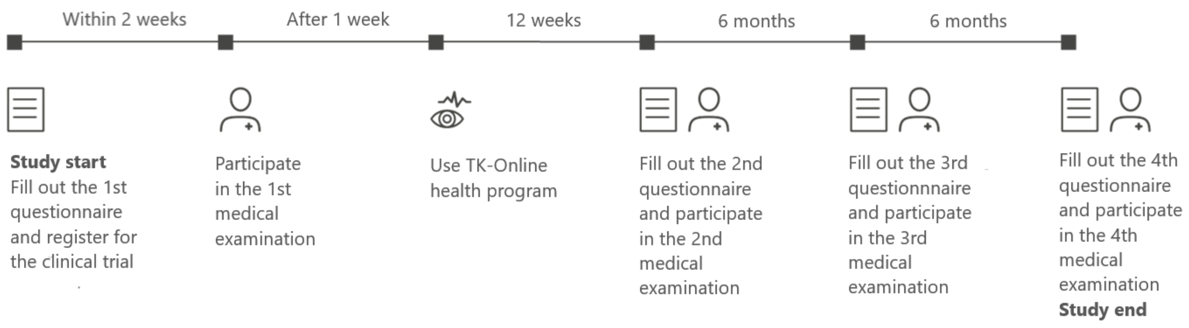

The following examinations are carried out on each of the four examination dates:

Anamnesis

In the form of an anamnesis, current sensitivities, general habits and disorders (e.g., alcohol, nicotine, sleep, allergies), medication intake, and (pre-)illnesses are recorded, and the medical certificate of fitness for sport is checked, and possible events are documented.

Physical activity behavior

Physical activity behavior is analyzed based on steps taken, time spent sitting, and physical activity performed. The Fitbit Charge 3™ activity tracker, which you will receive as part of the first medical examination, is used to record these. You will be asked to wear this activity tracker during the examination period. In addition, you will complete a questionnaire on physical activity behavior, the International Physical Activity Questionnaire (IPAQ).

Dietary behavior

To examine dietary behavior, you carry out a 7-day nutritional protocol with the NutriGuide® software. The energy, nutrient, and food intake are analyzed. You will receive an introduction to the nutritional protocol by the study staff at the first medical examination.

Physical fitness

Physical fitness is tested based on endurance and strength. To assess endurance, you will complete the Cooper 12-minute run test. Before this test, you will be given a standardized meal. The strength assessment is done using a leg and hand strength measuring device.

Body measurements

With regard to physical characteristics, body weight, body height, body mass index (BMI), body composition (fat mass, muscle mass), and waist circumference are determined. The waist circumference is determined with a measuring tape, and all other parameters are determined with a bioelectrical impedance analysis (BIA) scale.

Blood pressure, blood values, and vascular function

Blood pressure is measured via diastolic and systolic blood pressure using an electronic device. Blood values (total cholesterol, LDL cholesterol, HDL cholesterol, triglycerides, fasting

plasma glucose, HbA1c) are recorded by blood uptake (approx. 15 ml). Vascular function is measured non-invasively via an upper arm cuff by the electronic AngioDefender™ system.

The medical examinations will take about 4-5 hours per examination date. All medical examinations will be carried out at the Department of Sport and Sport Science (DoSS) of the University of Freiburg under standardized conditions and in delimited premises to protect privacy. The examinations that require a medical qualification (anamnesis, blood uptake) will be performed by the principal medical investigator of the study. The other examinations are carried out by the study director and the scientific study staff. After completion of the trial, you will receive a detailed final trial report with all your examination results.

Possible risks of the clinical trial

Concerning the health programs, muscle soreness may occur in the following days depending on the training condition and the training design. However, this is only temporary and does not have any disease value. Within the examinations, irritation or infection of the puncture site can occur in rare cases after blood uptake. There are no other risks.

Premature termination of the clinical trial

We would like to expressly point out the point that your participation in the clinical trial is voluntary. Your willingness to participate can be revoked at any time, even without giving reasons, and you can stay away from the medical examinations. If you do not wish to participate, you do not need to confirm the declaration of consent on the following page. If you wish to terminate your participation prematurely, you can contact the company Vilua Healthcare GmbH (Tel.: +49 30 40 740 655; e-mail: tk-studie@vilua.de) or the contact person, Ms. Judith Brame, at the Department of Sport and Sport Science (DoSS) of the University of Freiburg (Tel.: +49 761-203-54087, e-mail: tk-studie@sport.uni-freiburg.de) at any time by telephone or e-mail, stating your user name. In addition, you can also terminate your participation in the clinical trial early in the personal profile of your health program. In general, Vilua Healthcare GmbH is responsible for the technical implementation of the health programs and the administration of the consent forms and personal data. If you have any questions regarding the content of the clinical trial, please contact Ms. Judith Brame, Department of Sport and Sport Science (DoSS) of the University of Freiburg. In order to maintain anonymity vis-à-vis the staff, even in the event of queries regarding the content, please ensure that you do not express any information from which your person or your participation in the clinical trial could be inferred. The division of administrative and content-related processing among different institutions protects your person and strictly separates personal and research data. In principle, it is also possible that your participation in the trial may be terminated prematurely by the principal medical investigator of the study, e.g., if health impairments occur that partially or entirely exclude your ability to participate.

In the event of premature termination of your trial participation, all personal data already provided (first name/last name, telephone number), as well as your declaration of consent, will be deleted. Retroactive deletion of your research data (i.e., results of the medical examinations) is not possible. If you terminate your participation in the trial prematurely, your previous research data will be used in anonymized form for scientific evaluation. Neither participation nor non-participation in the clinical trial will result in any disadvantages for you.

Travel accident insurance

For the medical examinations, you are insured in the form of a travel accident insurance via ECCLESIA mildenberger HOSPITAL GmbH for all accidents that occur on the direct route from the place of residence to the place of the medical examinations (Department of Sport and Sport Science (DoSS) of the University of Freiburg). We expressly point out the point that this insurance cover does not apply if the average duration of the journey is extended or the journey itself is interrupted by purely private measures.

Participation in other research projects

During your trial participation, you must not participate in any other research project on physical activity and/or dietary behavior. Further participation may influence this trial.

Important information about the clinical trial

In order to be able to carry out the examinations in a standardized manner, you must appear fasting for all four examinations. This means that your last meal (including water, coffee, tea, etc.!) must have been at least 12 hours ago and your last alcohol consumption and exercise at least 48 hours ago. You should also get enough sleep the night before the examination. Furthermore, we ask you to bring sports clothes/shoes (indoor/outdoor) and showering things to all examinations. If you have any health impairments (at rest/under stress) or illnesses, we ask you to bring a current medical certificate of fitness for sport to the first examination.

Confidentiality, data protection, data management, and contact possibility

Personal data will be collected in the clinical trial. The GDPR-EU results in specific requirements for processing this data, which we would like to inform you about here (Art. 12 ff. GDPR). In particular, you have the following rights regarding the processing of personal data:

Right of access

You have the right to request information about your personal data processed in the clinical trial (Art. 15 GDPR-EU) and to receive a free copy of this data. This obligation to provide information includes, among other things: Purpose of data processing, categories of data, recipients or categories of recipients, duration of storage, right to rectification, deletion and objection, right of complaint to a supervisory authority, origin of data.

Purpose of the data processing

The legal basis for data processing is the declaration of consent for the clinical trial. Therefore, we would like to inform you about the purpose of data collection and processing.

The purpose of the clinical trial data collection is exclusively in scientific interest. The data serve to test the scientific effectiveness of various web-based health programs of the Techniker Krankenkasse from a sports and nutritional medicine perspective. To this end, quantitative data will be collected and processed by the principal medical investigator of the study, the study director, and the scientific study staff within four examinations at the Department of

Sport and Sport Science (DoSS) of the University of Freiburg. Without collecting and processing these data, the web-based health programs of the Techniker Krankenkasse cannot be meaningfully evaluated from a sports and nutritional medicine perspective.

Right to rectification of data

You have the right to request rectification of your personal data if it is incomplete or incorrect (Art. 16 GDPR-EU).

Right to restriction (blocking) of processing

You have a right to restrict processing (Art. 18 GDPR-EU) if one of the following applies:

- if the accuracy of the data is disputed by the data subject,
- if the processing is unlawful,
- if the purpose of the processing has ceased to exist, but the data are necessary for the assertion of legal claims of the data subject, or
- if there is an objection by the data subject following Art. 21 GDPR-EU.

Right to object

You have the right to object to the processing of your personal data (Art. 21 (6) GDPR-EU).

Right of withdrawal (right of withdrawal under data protection law)

You have the option to revoke your consent to the processing of your personal data at any time without giving reasons (Art. 7 (3) GDPR-EU). This does not affect the lawfulness of the processing of your personal data that took place until the revocation.

Right to data deletion

You have a right to have your personal data deleted (Art. 17 GDPR-EU).

If you wish to claim one of these rights, you can contact the data controller of the clinical trial.

Data responsibility

The person responsible for the data processing of the clinical trial is the study director at the Department of Sport and Sport Science (DoSS) of the University of Freiburg:

Judith Brame
University of Freiburg
Department of Sport and Sport Science
Schwarzwaldstraße 175
79117 Freiburg
Tel: +49 761-203-54087, e-mail: judith.brame@sport.uni-freiburg.de

If you have any questions about data protection, you can also contact the following office:

- Data Protection Officer of the University of Freiburg:

  Mr Klaus Scharpf, e-mail: datenschutz@uni-freiburg.de

Right of appeal

In addition, you have the right to complain if you believe that the processing of personal data concerning you is in breach of the GDPR (Art. 77 GDPR-EU).

- At the higher-level competent supervisory authority for the university:

  Home address:
  State Commissioner for Data Protection and Freedom of Information
  Königstrasse 10 a
  70173 Stuttgart

  Postal address:
  State Commissioner for Data Protection and Freedom of Information
  PO Box 10 29 32
  70025 Stuttgart

  Tel.: +49 711/61 55 41 - 0
  Fax: +49 711/61 55 41 - 15
  E-mail: poststelle@lfdi.bwl.de
  Internet: http://www.baden-wuerttemberg.datenschutz.de

Further notes

Data collection, processing, storage, transmission, and analysis of the clinical trial are anonymized. This means that all data directly identifying the person (e.g., first name/last name) are replaced by a pseudonymized code. The allocation of person and code is only possible via an allocation list, which is kept locked, secure, and separate from the coded research data. This list may only be viewed by the study director and the study staff. After written information on data collection following the European Union General Data Protection Regulation, they must maintain confidentiality by signature. This means that the allocation of the person and the clinical trial research data is only possible for these persons.

The pseudonymized research data of the clinical trial will be passed on exclusively to the Section of Health Care Research and Rehabilitation Research (SEVERA) of the Medical Center of the University of Freiburg for the sole purpose of data analysis. In this process, the research data of the clinical trial and the online trial are merged. Your declaration of consent gives your consent to this. You can object to this data transfer at any time without giving reasons. However, this will mean that you will no longer be able to participate in the clinical trial. The publication of the research data of the clinical trial is exclusively anonymized, i.e., in a form that does not allow any conclusions to be drawn about your person.

Concerning data deletion within the clinical trial, we have to inform you that all personal data will be deleted three years after completing the clinical trial (expected autumn 2024). From this point on, re-identification of the data is no longer possible. This procedure achieves a “de facto anonymization” of the research data (cf. recital 26 GDPR-EU, § 13 para. 2 SDPA BW). After ten years, all research data (i.e., results of medical examinations) are finally deleted. This results in the complete deletion of all data collected in the clinical trial.

Responsible for the clinical trial

Department of Sport and Sport Science (DoSS) of the University of Freiburg, Schwarzwald-straße 175, 79117 Freiburg.

Contact persons: Prof. Daniel König, MD

Ms. Judith Brame (Tel: +49 761-203-54087)

E-mail address tk-studie@sport.uni-freiburg.de

We thank you very much for your support!
