# Supplementary material for: Health Effects of a 12-Week Web-Based Lifestyle Intervention for Physically Inactive and Overweight or Obese Adults: Study Protocol of Two Randomized Controlled Clinical Trials
Source: Int J Environ Res Public Health. 2022 Jan 26;19(3):1393. doi: 10.3390/ijerph19031393 (PMC8835149; doi:10.3390/ijerph19031393)
Supplement: Supplementary file 1 [file ijerph-19-01393-s001.zip › File S3.pdf]

Albert-Ludwigs-Universität Freiburg

Research project

Evaluation of web-based health programs of Techniker Krankenkasse (Fitness)

Clinical trial

DECLARATION OF CONSENT

I hereby confirm that I have received an information letter explaining the objectives, contents, and data protection issues of the clinical trial. I had sufficient time to think about it, and all my questions were answered satisfactorily. The clinical trial is being conducted, coordinated, and supervised by the Department of Sport and Sport Science (DoSS) of the University of Freiburg.

My trial participation is voluntary. I have been informed that I have the right to revoke this consent in whole or in part without giving reasons and to discontinue trial participation at any time. In this case, all personal information I have already provided (first name/last name, telephone number), as well as my declaration of consent, will be deleted. Retroactive deletion of my research data (i.e., results of the medical examinations) is not possible. If my participation is terminated, my research data will be included in the scientific analyses in anonymized form. I will not suffer any disadvantages from participation or non-participation.

I have been informed of my data protection rights following the European Union General Data Protection Regulation. I agree to collect, process, store, forward, analyze and publish my data by these data protection rights within the clinical trial. I am informed that I can object to data transfer at any time and without giving reasons. However, this will mean that I will no longer be able to participate in the clinical trial. The data of the clinical trial will be analyzed by the Section of Health Care Research and Rehabilitation Research (SEVERA) of the Medical Center of the University of Freiburg and the Department of Sport and Sport Science (DoSS) of the University of Freiburg. The results of the clinical trial are presented in anonymized form.

Personal data or information that allows conclusions to be drawn about my person will not be passed on to third parties or published. All personal data will be deleted three years after completion of the clinical trial (probably in autumn 2024). After ten years, all research data (i.e., results of the medical examinations) will be deleted. All data will be treated as strictly confidential and used exclusively within the purpose of the clinical trial.

Under the conditions listed here and in the study information, I agree to participate in the clinical trial. My contact details (first name/last name, telephone number) and information on availability entered on the following page may be passed on to the Department of Sport and Sport Science (DoSS) of the University of Freiburg to arrange appointments. My declaration of consent and the study information are automatically stored in the personal area of my health program and can be viewed and, if necessary, revoked by me there at any time.
